# Mr. Richmond, on Ischuria

**Published:** 1800-10

**Authors:** Thomas Richmond

**Affiliations:** Paisley


### *To the Editors of the Medical and Phyſical Journal.*

GENTLEMEN,

ENCOURAGED by your reception of my firſt Eſſay in Surgery, permit me to addreſs you with a few thoughts thrown together on ſome caſes in that branch of ſcience, which have lately fallen under my care, chiefly relative to inflammation and abſceſs of the bladder. All the cavities of the body, as well as its other parts, are known to be liable to theſe affections, and have already been ably treated of by practical authors; ſtill, I think, from the following caſe which I am about to delineate, we muſt proceed a ſtep further, and aſcertain the time and circumſtances which will warrant us to interfere in it by Surgery, after the effects of medicine have been tried in vain.

Allen Green, aged 40, came on the Diſpenſary here in March laſt, labouring under a temporary ſtoppage of urine, which, when voided, depoſited purulent matter in conſiderable quantity; tongue, pulſe, and other ſymptoms indicating a febrile ſtate of the ſyſtem. He attributed his complaints to an imprudent uſe of injections for a gonorrhœa, which he had contracted ſome months before. The pain in the region of the bladder was conſtant, but aggravated on preſſure, or on the return of the inclination to make water, which was now become very very frequent, and could not be effected without fevere ftraining, and even, at times, not without the affiftance of the bougie or catheter. Upon the introduction of thefe, the ftricture appeared to be entirely at the neck of the bladder, behind the proftrate gland; but upon examination of that organ, no fwelling or difeafe could be perceived. It was evident, in this ftage of the difeafe, that the time was paft to prevent or cure the complaint by bleedings or other antiphlogiftic remedies, which, to be fuccefsful, muft be ufed early, and in due proportion.—All that could be done, therefore, was to procure a regular evacuation of the purulent matter along with the urine, and try the ufe of internal remedies. The ufual remedies were prefcribed, gentle diuretics, uva urfi, opiates, laxatives, tepid bath, mild diet, &c. with the ufe of the bougie. Thefe he could not endure to perfevere in; they rather feemed to do harm.

In employing thefe remedies, time paffed away, and his general health was evidently far on the decline, while the local complaint continued unabated; from which circumftances it was become apparent, the patient would fall the flow but certain victim of hectic difeafe; an event that had occurred to two or three other patients labouring under fimilar fymptoms, that had fallen within my obfervation in the courfe of practice; and I began to think, how far I was warranted to proceed by incifion to cut acrofs the ftricture. My idea was to perform the incifion as if for the lateral operation, to preferve the wound open, and wafh out the bladder with warm milk and water.—The patient was apprifed of his fate, and confented to fubmit to whatever might be deemed compatible with life. Before proceeding to fuch an operation, I deemed it reafonable to have the affiftance of a confultation; when, after perufing the moft approved furgery, examining the patient, and weighing his prefent fituation, his legs being œdematous, and his ftrength much impaired, it was judged improper to operate. The event was forefeen, and the patient died.

Upon diffection, the body exhibited the ufual emaciated ftate. The great inteftine was flightly inflamed all over the arch of the colon; two or three intus-fufceptions were met with. The bladder, upon being cut through, was found in a thickened ftate, and full of fœtid and clotted pus. There was no ftricture in the urethra till the very entering of the bladder; and it feemed to have arifen there, in a great meafure, from the exceffive thickening of its coats. It was then that I regretted that I had not feized the opportunity earlier, and have confidered the ftricture as a fufficient *warrant*, by a full and free incifion either by the knife or gorget, to have laid open the bladder, bladder, and thus have given Nature, with the affiftance of art, a chance to have overcome the difeafe; and to lay it down in future, that whenever a ftricture occurs, irremovable by bougies, accompanied with a purulent difcharge, to proceed as we would do in other cafes of abfcefs,—to operate freely, and give full vent to the matter.

I was much confirmed in the above reafoning, from obferving the phaenomena which occurred in a cafe of deep-feated femoral hernia, in a female patient, that fell to my fhare in practice at the fame time I was treating the above. When called to the patient with hernia, a poor woman refiding in the country, the tumor had been down nine days; and from her own efforts to reduce it, was become exceedingly painful and indiftinct. It was evident from the aggregate of the fymptoms, as well as the length of time they had been prefent, that the ftate of the tumor and fate of the patient were very critical. An extenfive incifion was made acrofs the tumor through the outward fkin; the fac was cautioufly fcratched: but it was not till after cutting through a confiderable depth of fat and glandular fubftance that a fluctuation was felt; at length an enormous quantity of fœtid pus burft out. The bag was laid extenfively open, its different laminæ being quite indiftinct; and after wiping the wound, the inteftine was found deep in the bottom, in a gangrenous ftate. The patient was put to reft through the night, not without anxiety for her fate. In the morning fhe was found confiderably relieved, and the fever much abated. The ftricture had been fairly divided, and the pus evacuated. Nature proceeded to do the reft, to throw off the floughs both in the inteftine and membranes, and to heal the wound, which is now nearly clofed; which leads me to keep in view the practice, in regard to the firft cafe mentioned in this Effay, and to extend the propofition in Surgery, That whether the *ftricture* arifes from external wounds or difeafe, whether the caufe of irritation is foreign or generated matter, it is our bufinefs, when neceffity demands it, to cut through the one, in order that we may relieve the fyftem from the other. I remain,

Gentlemen,

Your moft obedient,

THOMAS RICHMOND.

*Paisley,*

*Aug.* 30, 1800.

